# The Intraventricular Pseudocyst as a Complication of Ventriculoperitoneal Shunt: A Rare Case Report and Review of Literature

**DOI:** 10.7759/cureus.20546

**Published:** 2021-12-20

**Authors:** Aldo José Ferreira da Silva, Fernando E Castro Pinheiro Gomes, Suely C De Lima

**Affiliations:** 1 Pediatric Neurosurgery Division, Santa Mônica Teaching Maternity-Alagoas State University of Health Sciences, Maceio, BRA; 2 Division of Pediatric Neurosurgery, General State Hospital (GSH), Maceio, BRA; 3 Division of Anesthesiology, General State Hospital (GSH), Maceio, BRA

**Keywords:** ventriculoperitoneal shunt, endoscopic, computed tomography, pseudocyst, hydrocephalus

## Abstract

Ventriculoperitoneal shunting is the most common treatment for hydrocephalus. Various complications can occur, including the formation of a pseudocyst. Reviewing the literature, we report a rare case of intraventricular pseudocyst as a complication of ventriculoperitoneal shunt in a child. A seven-month-old child with a ventriculoperitoneal shunt presented with a large intraventricular cyst on computed tomography of the skull. It was decided to remove the ventriculoperitoneal shunt, perform an endoscopic fenestration of the pseudocyst, and place an external ventricular shunt. After 14 days of antibiotic treatment, a new ventriculoperitoneal shunt was placed. The child grew up with delayed milestones and epilepsy. Pseudocysts may be a possible complication of ventriculoperitoneal shunting. It is rare for pseudocysts to be located inside the ventricle, as in the present case; the pathophysiology is unclear, and the child can have sequelae after treatment.

## Introduction

Ventriculoperitoneal shunting is the most common form of drainage of cerebrospinal fluid in the treatment of hydrocephalus, especially in childhood [1]. The incidence of complications after placement of a ventriculoperitoneal shunt ranges from 24% to 47% [2]. In children in the first year of placement, an estimated failure was 40%-50% [3]. Complications of ventriculoperitoneal shunt can be divided into mechanical (proximal or distal obstruction, valve malfunction, disconnection, and total migration of the shunt) and nonmechanical (infection, abdominal pseudocyst, valve insufficiency, trapped fourth or lateral ventricle, subdural hemorrhage, and overdrainage) [4]. In children younger than two years, the main problems with shunt are obstruction (mainly distal and proximal) and infection [5].

This is the report of a case of intraventricular pseudocyst, a rare complication after placement of a ventriculoperitoneal shunt in a child.

## Case presentation

**Figure 1:**
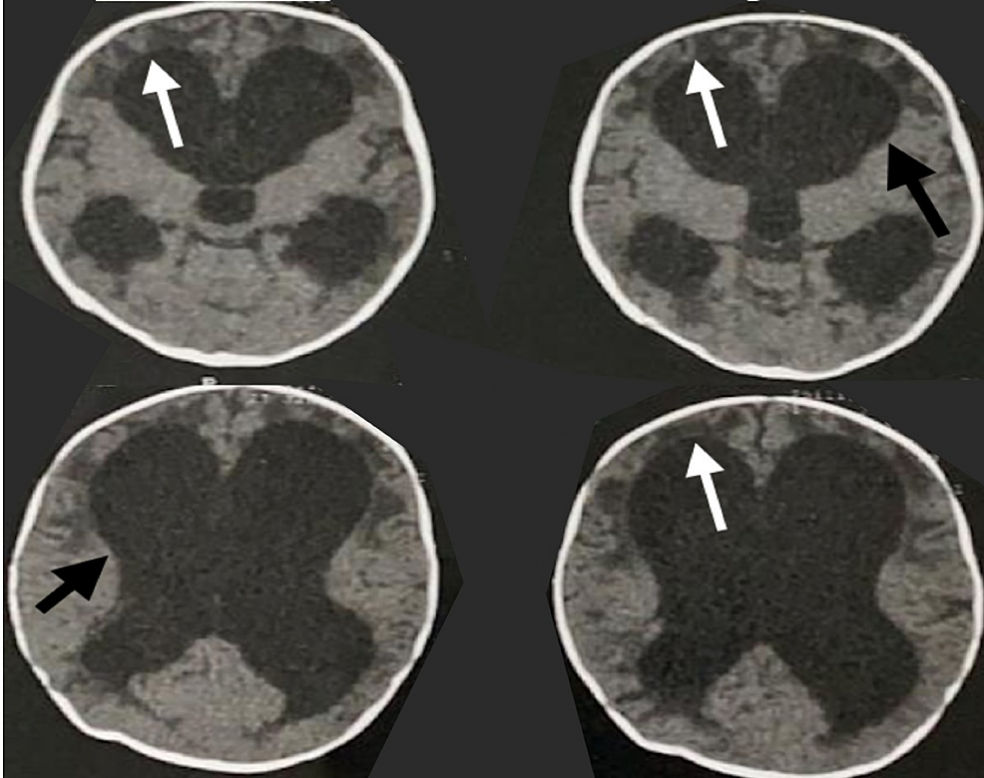
Axial computed tomography (CT) scan Shows enlargement of the ventricles (black arrows) with transependymal edema (white arrows).

**Figure 2:**
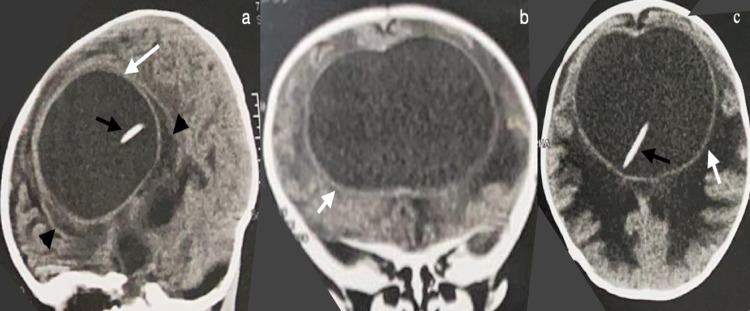
Computed tomography (CT) scan without contrast enhancement CT scan without contrast enhancement in the sagittal (a), coronal (b), and axial (c) planes. Shows pseudocyst (white arrows) inside the ventricular cavity (black arrowheads) and the catheter (black arrows).

**Figure 3:**
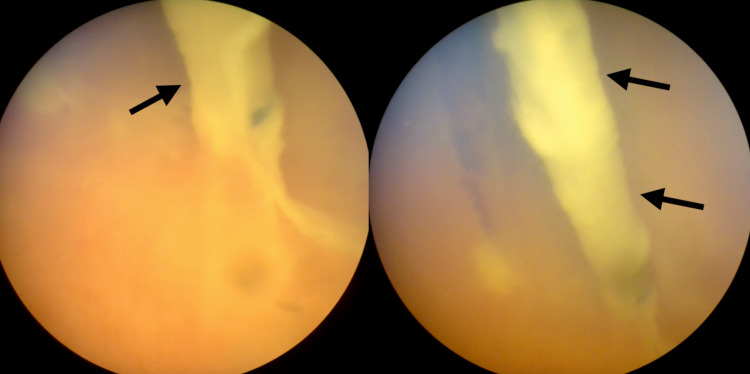
Endoscopic image Showing ventricular catheter inside the cystic cavity (black arrows).

The patient was born after 39 weeks of gestation by cesarean section, with Apgar scores of 8 and 8 at one and five minutes, respectively, weighing 3,100 g, with a height of 46 cm and a head circumference of 49.5 cm. The mother had no history of infection during pregnancy or consanguineous marriage. A diagnosis of hydrocephalus was established after computed tomography of the skull (Figure 1) was performed. It was then decided to place a ventriculoperitoneal shunt. After seven months, the child was admitted to the emergency room with fever, irritation, excessive crying, and a bulging and tense anterior fontanelle. Computed tomography of the skull showed a large intraventricular cystic lesion (Figure 2a-2c). The procedure involved removing the ventriculoperitoneal shunt (no obstruction in the distal catheter was visualized) (Figure 3), then performing an endoscopic cyst fenestration, and placing an external ventricular shunt. The content of the cystic lesion was cerebrospinal fluid (CSF), with the following results: glucose 2.24 mmol/L, protein 17.61 g/L, a cell count of 66.6/mm^3^ (1% polymorphonuclear, 99% monocytes), and the culture was negative. Prophylaxis with cefazolin sodium 3 g/day was given for five days, and a new ventriculoperitoneal shunt was placed with no further complications. At a one-year follow-up, the child grew with delayed milestones and epilepsy, but the ventriculoperitoneal shunt remained functional.

## Discussion

The formation of pseudocysts is a rare complication of ventriculoperitoneal shunting. These pseudocysts are most frequently located in the abdomen, but there are reports of other sites, such as subcutaneous, in the diploe, and intraparenchymal [6-9]. In the presently reported case, the pseudocyst was located at an unusual site, namely within the ventricle.

In 1954, Harsh was the first to describe an abdominal pseudocyst [10]. Abdominal pseudocysts correspond to about 0.25%-10% of all complications of ventriculoperitoneal shunts [11]. Several causes can explain the formation of these abdominal pseudocysts: (1) infection; (2) increased protein in the CSF; (3) liver dysfunction; (4) allergic reaction to the shunt material; (5) previous abdominal surgery or multiple revisions of the shunt in the abdomen; and (6) history of intracranial tumors [11]. Hahn et al. describe that 80% of pseudocysts are caused by infection [12]. All these causes eventually favor the formation of a fibrous tissue cavity or an inflamed serous surface, without epithelial wall coating (hence the name “pseudocyst”) and filled with CSF and debris [6]. In the specific case of the formation of intraparenchymal pseudocysts, another possible explanation would be that the pseudocyst results from a distal blockade of the peritoneal catheter, which causes an increase in hydrostatic pressure, thereby forcing the CSF to flow from the ventricular system into the interstitial space, forming the pseudocyst [9].

In the presently described case of an intraventricular pseudocyst, some possible causes could be infection, increased protein in the CSF, or an allergic reaction to the shunt material. Since the distal peritoneal catheter was not blocked, the formation of the pseudocyst cannot be explained by the same mechanism by which intraparenchymal pseudocysts are formed, according to the literature.

## Conclusions

Several complications can occur in children after a ventriculoperitoneal shunt placement, especially in the first year. A less frequent complication is the pseudocyst formed in the abdomen, although there are other locations, such as intraventricular.

Intraventricular pseudocysts are rare, and their pathophysiology is not well clarified. Unlike abdominal pseudocysts, intraventricular pseudocysts can cause sequelae in developing children after their resolution.
